# Determination of sialic acid in serum samples by dispersive solid-phase extraction based on boronate-affinity magnetic hollow molecularly imprinted polymer sorbent†

**DOI:** 10.1039/c9ra00511k

**Published:** 2019-02-12

**Authors:** Wei Huang, Xingyu Hou, Yukui Tong, Miaomiao Tian

**Affiliations:** Key Laboratory of Photochemical Biomaterials and Energy Storage Materials, College of Chemistry and Chemical Engineering, Harbin Normal University Harbin 150025 China mmttqqq@163.com

## Abstract

Boronate-affinity magnetic hollow molecularly imprinted polymers (B-MhMIPs) were prepared with sialic acid (SA) as the template, 3-aminophenylboronic acid (APBA) as the functional monomer and glycidilmethacrylate (GMA) as the co-monomer to chemisorb Fe_3_O_4_ nanoparticles. Furthermore, the hollow structure made the nanoparticles have more binding sites at both internal and external surfaces, which can facilitate the removal of template molecules from polymers and enhance the adsorption abilities towards SA. After optimizing, the adsorption pH was controlled at 4.0, and this was different from most *cis*-diol-containing compounds. Under the optimal conditions, the limit of detection for SA was 0.025 μg mL^−1^ (*n* = 3). This method was applied to analyze serum samples with different spiked levels, and the recoveries of the SA were in the range of 70.9–106.2%. These results confirmed the superiority of the B-MhMIPs for selective and efficient enrichment of trace SA from complex matrices.

## Abstract 2

Boronate-affinity magnetic hollow molecularly imprinted polymers were prepared with sialic acid (SA) as a template to selectively extract SA from serum samples coupled with HPLC-UV.

## Graphical abstract

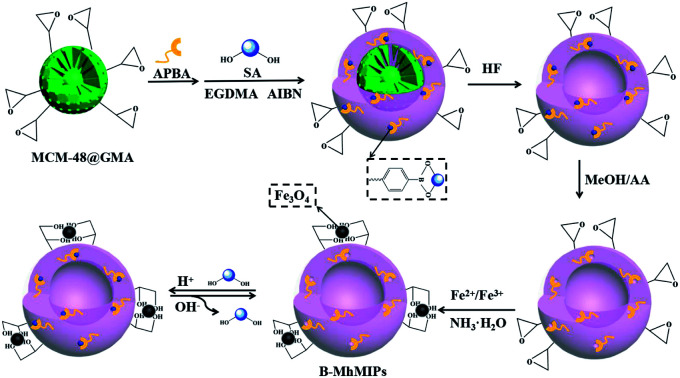

## Introduction

Molecularly imprinted polymers (MIPs) are an adsorbent material, containing specific recognition sites complementary in size, shape, and chemical functionality with the template molecules.^1,2^ In addition, MIPs also possess thermal stability, physical robustness and desirable selectivity, and are wisely applied in the selective extraction of target substances from varied and complex matrices (such as food, traditional Chinese medicine, biological and environmental samples).^3–8^ However, the deeply embedded recognition sites in highly cross-linked polymer networks decreased the binding capacity. Therefore, hollow-molecularly imprinted polymers (h-MIPs) have attracted extensive attention owing to their larger specific surface area, higher utilization ratio of binding sites, and faster mass transfer. Generally, h-MIPs are core–shell structures, and the core can be etched or dissolved by acids or organic solvents to form a hollow structure. Thus, the binding sites can be fully used at the external and internal surface.^9^ Nowadays, magnetic molecularly imprinted polymers (MMIPs) have further advantages that they not only can achieve fast solid–liquid separation by an external magnetic field within a few seconds, avoid complex solid-phase extraction (SPE) operation, but also maintain the advantage of MIPs.^10,11^ The MMIPs also possess highly selective binding characteristics to the target template and homologues, and are beneficial for the process of pre-treatment with samples.^12^ Therefore, in recent years, the MMIPs have been widely used for selective adsorption of target substances from complex matrices.^13^ And also, the hollow and magnetic molecularly imprinted technologies must exhibit a great potential for the selective extraction of small molecules or other target compounds.

SA, a kind of monosaccharide with a nine-carbon backbone, is typically found at terminal position of glycoconjugates.^14^ SA plays an important role in physiological and pathological processes. The serum is the most important human biofluid containing SA and especially valuable in clinical diagnosis of several diseases.^15^ Gruszewska *et al.*^16^ have described the marker capability of SA in serum for diagnosis and evaluation the location of tumor in patients with primary pancreatic cancer. So the measurement of serum SA can be valuable in earlier diagnosis of malignant disease or monitoring the tumour bulk in response to treatment.^17^ In addition, the levels of SA in serum can indicate a malfunction of many organs of the human body and also indicate the progression and spread of human malignancies.^18^ Several human genetic disorders such as Salla disease and myopathy are associated to disorder of SA levels. SA is a general term for a class of substances, which the most common member is *N*-acetylneuraminic acid (Neu5Ac).^19^ Moreover, Neu5Ac is the major form of SA in higher animals including mammalians and a few microorganisms, because of its involvements in the mediation and many specific biological functions.^20^ Clearly, the detection for SA as a biological marker substance is arousing a great of attention and becoming crucial. In the present work, SA will refer to the Neu5Ac.

It is therefore obvious that quantitative and qualitative analysis of SA from complex serum products is considerably important. Chromatographic methods, especially for high performance liquid chromatography (HPLC), are considered as the powerful analytical techniques for complex system. Prior to detection, derivatization with a chromophore or fluorophore for sensitive detection is needed. Among the derivatization reagents for the determination of SA in human serum, 1,2-diamino-4,6-dime-thoxybenzene (DDB) has been frequently used by HPLC.^21,22^

Despite substantial technological advances in the analytical field, HPLC cannot handle relatively complex samples directly. As a result, sample preparation is a critical step for analysis of trace compounds in complicated sample matrix. In order to decrease the matrix effect and increase the detection sensitivity, many preconcentration methods have been reported for serum SA. It is worth mentioning that, with the borate groups as the functional monomer, the MIPs can exhibit high affinity and great selectivity in the separation of *cis*-diol molecules (nucleosides, glycoproteins, saccharides and other *cis*-diol-containing compounds) from complex biological samples.^23,24^ GMA has an epoxide ring that offer fewer potential hydrogen bonds compared with the free hydroxyl group, which have less interfere in MIPs formation.^25^ Furthermore, the epoxide ring can be opened to form 1,2-diol groups. Therefore, GMA is always used as pro-hydrophilic co-monomer for preparation of hydrophilic MIPs.^26^ Interestingly, Wan and co-workers^27^ have reported that poly(glyceryl monomethacrylate) can be chemisorbed onto Fe_3_O_4_ nanoparticles surface through their 1,2-diol groups by forming five-membered chelate rings with the Fe atoms. Based on this, Li and co-workers^25^ have successfully prepared hollow porous molecularly imprinted polymers by introducing GMA as co-monomer, and MCM-48 as sacrificial support. After that, epoxide ring of GMA has been opened for chemisorbing Fe_3_O_4_ nanoparticles to prepare magnetic hollow molecularly imprinted polymers (MhMIPs). Accordingly, we speculate that MhMIPs can be synthesized by using GMA as co-monomer to immobilize Fe_3_O_4_.

Due to the *cis*-diol structures of SA, which can covalently bind with borate groups and generate a steady five-member cyclic esters.^28^ Therefore, we presented a new process for synthesis B-MhMIPs, which used SA as template, MCM-48 as sacrificial support, APBA was selected as the boronate-affinity functional monomer and GMA as co-monomer. And the epoxide ring of GMA was opened with ammonium hydroxide (NH_3_·H_2_O, 28%, v/v), the magnetic Fe_3_O_4_ nanoparticles were attached *via* a simple co-precipitation process. The synthesized nanoparticles were characterized, and the experimental conditions were also optimized. The resulting materials can specific extract SA under acidic conditions and release under alkaline conditions, this result was different from adsorption properties of other *cis*-diol-containing compounds.^23^ In addition, competitive adsorption, and adsorption capacity for real samples were also investigated. These results indicated that the B-MhMIPs adsorbents showed high binding capacity, fast binding abilities and excellent selectivity.

## Experimental

### Materials and chemicals

SA, ethylene glycol dimethacrylate (EGDMA), *o*-phenylenediamine (OPD), and APBA were purchased from Aladdin Reagent (Shanghai, China). Analytical grade ethanol (EtOH), acetonitrile (ACN), and methanol (MeOH) were purchased from Beijing Chemical Works (Beijing, China). Azobisisobutyronitrile (AIBN) was purchased from Tianjin Chemical Plant (Tianjin, China), and was purified by recrystallization from EtOH and dried under vacuum at room temperature prior to use. Tetraethyl-orthosilicate (TEOS), ferric chloride hexahydrate (FeCl_3_·6H_2_O), and ferrous chloride tetrahydrate (FeCl_2_·4H_2_O) were purchased from Sinopharm Chemical Reagent (Shanghai, China). Cetyltrimethyl ammonium bromide (CTAB), NH_3_·H_2_O, hydrofluoric acid (HF) and acetic acid (AA) were provided by Shanghai Chemical Reagent (Shanghai, China). GMA and tetrahydrofuran (THF) were supplied by Energy Chemical (Shanghai, China). HPLC grade MeOH and ACN were supplied by Fisher Scientific (New Jersey, USA). All other reagents were bought from various commercial sources and were of analytical or HPLC grade. NdFeB magnets were purchased from Yingke (Beijing, China) with a cuboid shape of 15 mm × 10 mm × 3 mm and a surface magnetic field of 3000 G.

The stock solution of 1000 μg mL^−1^ SA was prepared in MeOH and stored at 4 °C in the refrigerator. The standard working solutions were prepared in water from the stock solutions to obtain the required concentrations prior to use. The rabbit serum samples were diluted 50-fold by NaH_2_PO_4_ buffer (pH 4.0), and then were subjected to the assay. Prior to the HPLC procedure, the sample solutions were filtered through 0.45 μm millipore filter to remove impurities.

### Apparatus

HPLC analysis was equipped with an LC-20A HPLC system (Shimadzu, Japan), which consisted of a liquid delivery pump (LC-20AT), an autosample injector (SIL-20A), a column oven (CTO-20A), an ultraviolet detector (SPD-20A), and the Labsolutions workstation (Shimadzu, Japan) for the acquisition and analysis of the chromatographic data. A syncronis C18 column (4.6 × 250 mm, 5 μm, Thermoscientific, USA) was employed for the chromatographic separation. A Millipore Milli-Q water purification system (Millipore, Bedford, MA, USA) was used to purify deionized water (DDW), and the DDW produced at 18.2 MΩ cm was prepared for sample solutions.

A PHSF-3F pH meter (Shanghai Precision and Scientific Instrument, Shanghai, China) was used for pH measurements. A ZK-82BB electric vacuum drying oven (Shanghai Experimental Instrument Co. Ltd., China) was utilized. A KQ-100E ultrasonic cleaner (Kunshan Ultrasonic Instruments Co. Ltd., China) was used to clean and disperse. An HZQ-F160A thermostatic oscillator (Shanghai Yiheng Technology Co. Ltd., China) was utilized. An LD5-2A centrifuge (Beijing Jingli centrifuge Co. Ltd., China) and an IKA RW20 mechanical stirrer (IKA Corporation, Germany) were used for centrifuging and stirring, respectively. A Model DOA-P504-BN pump (IDEX, USA) was used. A transmission electron microscope (TEM, JSM 2000-F, JEOL Company, Japan) was used to observe the surface morphology. The Fourier transform infrared spectra (FT-IR) were recorded with a Thermo Nicolet 670 FT-IR instrument (Thermo, USA). A Q500 thermal gravimetric analysis (TGA) system (TA Company, USA) was employed to investigate the thermal behavior of the adsorbents. The vibrating sample magnetometer (VSM, Lakeshore 7410, USA) was used to investigate the magnetic properties of the nanoparticles at room temperature. The X-ray diffractometer (XRD, Rigaku RINT 2500, Rigaku Corporation, Japan) was used to perform the identification of crystalline phase.

### HPLC analysis

The optimized mobile phases consisted of 8% ACN and 92% water containing 1% THF and 0.2% H_3_PO_4_ (v/v) at a flow rate of 0.9 mL min^−1^. The injection volume was 20 μL, and the column temperature was controlled at 35 °C. The preferential detection wavelength was 230 nm. Before use, the mobile phases were filtered through 0.45 μm millipore filter, and then degassed for 15 min.

Pre-column UV derivatization for detection of SA:^29^ 0.5 mL 10 mg mL^−1^ OPD solution was added to the 1 mL SA standard solution, and the reaction was allowed to proceed for 50 min at 80 °C, then cooling to room temperature. Before use, the solutions were filtered through 0.45 μm millipore filter.

### Preparation of B-MhMIPs

**Fig. 1:**
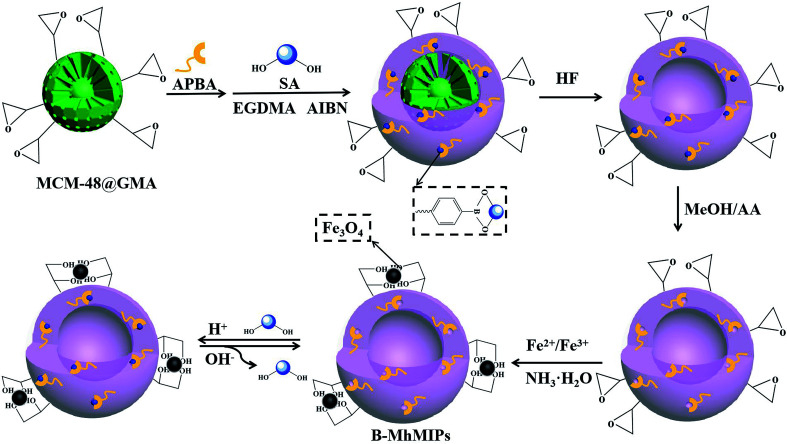
Illustration of the procedure for preparing B-MhMIPs nanoparticles.

The procedure to prepare B-MhMIPs is depicted in Fig. 1. At first, MCM-48 microspheres were synthesized under same condition described in our previous report.^9^ Then, 500 mg MCM-48 and 2.0 mmol GMA were dissolved in EtOH, stirred at 70 °C for 5 h and purged with N_2_ to prepare MCM-48@GMA. After that, the boronate-affinity hollow molecularly imprinted polymers (B-hMIPs) were prepared as follows. In the first stage, 0.15 g MCM-48@GMA and 0.10 g APBA were dispersed in 20 mL NH_3_·H_2_O, purged with N_2_, and stirred at room temperature for 2 h to prepare MCM-48@APBA. Then, 0.50 mmol SA and 500 mg MCM-48@APBA were dispersed in 40 mL ACN and stored at 4 °C for 12 h to form the preassembly solution. Finally, 10 mmol EGDMA and 0.15 g AIBN were added into above solution, and the reaction was allowed to proceed under constant stirring at 60 °C for 24 h.

After polymerization, boronate-affinity molecularly imprinted polymers were collected by filtration, rinsed with EtOH until the supernatant was clear. In the second stage, the hollow microspheres were fabricated by simply dissolving the MCM-48 matrix using HF/EtOH (1 : 9, v/v) under continuously stirring for 8 h. The products were washed repeatedly with a mixture of MeOH/AA (8 : 2, v/v) to extract the embedded template in a Soxhlet extractor until no SA was detected in the supernatant with a UV-vis spectrophotometer at 230 nm. Finally, B-hMIPs were dried under vacuum at 60 °C.

Finally, magnetic Fe_3_O_4_ was immobilized on B-hMIPs by co-precipitation process. Briefly, 200 mg B-hMIPs and 0.24 mmol FeCl_3_·6H_2_O were added into 60 mL water/MeOH (4 : 1, v/v), purged with N_2_, and then ultrasounded for 10 min. Then, 0.12 mmol FeCl_2_·4H_2_O was added into the above solution, purged with N_2_, and mechanically stirred at 80 °C for another 10 min. After that, 9 mL NH_3_·H_2_O was added dropwise into the system under constant stirring, and kept stirring at the same temperature for another 1 h. After being cooled to ambient temperature, the B-MhMIPs were collected by magnet, rinsed with EtOH, and then dried overnight under vacuum at 60 °C. For comparison, the boronate-affinity magnetic hollow non-imprinted polymers (B-MhNIPs) were also prepared in the same way, except that the template of SA was not added.

### Solid phase extraction operation procedure

Extraction process was carried out according to the following step: 10 mL 1 μg mL^−1^ SA sample solution (pH = 4.0) was placed in a 50 mL beaker, 20 mg B-MhMIPs/B-MhNIPs were added to sample solution, the solution was shaken at room temperature for 40 min to facilitate mass transfer and adsorption of the SA onto magnetic nanoparticles, after the adsorption of analytes, by applying a magnet, B-MhMIPs/B-MhNIPs were separated from the sample solution, and the supernatant was decantated and the colloid obtained was eluted with 10 mL 20 mmol L^−1^ pH 10.0 phosphate buffer solution by shaking for 10 min. Finally, the eluate was isolated from the sorbent by applying a magnet and 20 μL of eluate was derived and injected into the HPLC system for subsequent analysis. Due to the nano size of particles, it is necessary to filter the samples with 0.45 μm millipore filter before injection to HPLC.

### Adsorption experiment

The recognition and adsorption capacities of B-MhMIPs/B-MhNIPs to SA were investigated by conducting adsorption equilibrium experiments. The 20 mg B-MhMIPs/B-MhNIPs were dispersed in 10 mL of 1 μg mL^−1^ SA sample solution. After shaking at room temperature for 40 min, B-MhMIPs/B-MhNIPs were separated by magnet, the supernatant was collected and derived for HPLC determination after filtering with a 0.45 μm millipore filter.

Typically, the adsorption amounts, *Q* (μg g^−1^), were calculated by eqn (1),1
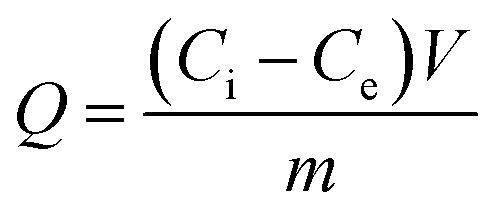
where *C*_i_ and *C*_e_ represent the initial concentration before adsorption and final concentration extracted with B-MhMIPs/B-MhNIPs (μg mL^−1^), respectively. *V* is the volume of solution (mL) and *m* is the amount of B-MhMIPs/B-MhNIPs (g).

The recognition abilities of B-MhMIPs/B-MhNIPs for SA were also evaluated by the imprinting factor (IF), which is defined as follows:2
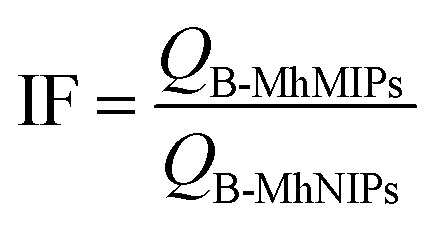
where *Q*_B-MhMIPs_ and *Q*_B-MhNIPs_ are the adsorption capacities of SA for B-MhNIPs/B-MhNIPs, respectively.

### Sample preparation

All experiments were performed in compliance with the relevant laws and institutional guidelines. The rabbit plasma were transferred into centrifuge tubes and frozen at −20 °C. The plasma samples were thawed at room temperature. The collected rabbit plasma samples were stored in separate glass tubes containing EDTA as an anticoagulant additive. A blank serum sample was prepared by deproteinization process in the plasma sample (2.0 mL) using 8.0 mL ACN. Before use, the rabbit serums were centrifuged at 10 000 rpm for 10 min at 5 °C. Then the supernatant were degassed and homogenized with an ultrasonic bath and filtered through 0.45 μm millipore filter prior to analysis.^19^

## Results and discussion

### Characterization of B-MhMIPs

**Fig. 2:**
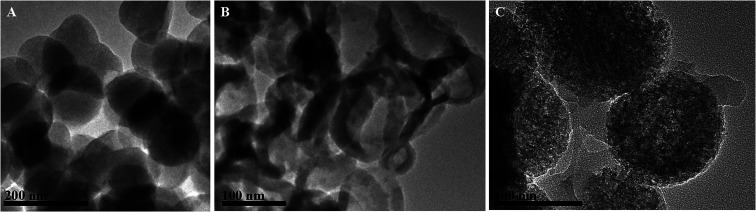
TEM images of MCM-48 spheres (A), B-hMIPs (B), and B-MhMIPs (C).

**Fig. 3:**
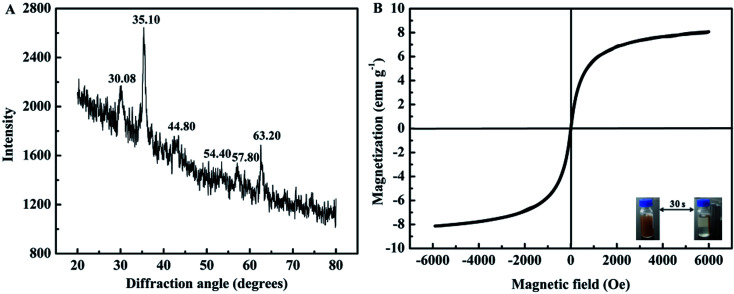
XRD (A) and VSM (B) curves of B-MhMIPs.

The B-MhMIPs were characterized by TEM, XRD, VSM, FT-IR and TGA. The TEM images of MCM-48, B-hMIPs and B-MhMIPs are shown in Fig. 2. The results indicated that the successful preparation of MCM-48 with smooth surface and good dispersion in Fig. 2A. The hollow structure in Fig. 2B demonstrated the removal of MCM-48. The hollow structure ensured the SA molecules entered into the imprinted sites from internal and external sides of the nanospheres, which led to fast binding and desorption. As can be seen from Fig. 2C, clearly presented the existence of Fe_3_O_4_ nanoparticles of the B-MhMIPs. The immobilization of Fe_3_O_4_ nanoparticles were also confirmed by the XRD and VSM (Fig. 3).

The XRD patterns of B-MhMIPs are shown in Fig. 3A. Six representative peaks of magnetite (JCPDS file no. 19-0629) at 30.08°, 35.10°, 44.80°, 54.40°, 57.80° and 63.20° were relatively discernible observed in the curve.^30^ Furthermore, Fig. 3B shows the magnetic property of B-MhMIPs measured by the VSM. The mass content of Fe_3_O_4_ nanoparticles can be confirmed by the relatively low magnetic saturation (8.2 emu g^−1^), however, the magnetic separation ability of B-MhMIPs was not seriously affected. As shown in the insets of Fig. 3B, in the absence of an external magnetic field, a brown homogeneous dispersion existed. However, the brown particles were attracted to the wall of vial and the dispersion became clear and transparent in the presence of an external magnetic field.

**Fig. 4:**
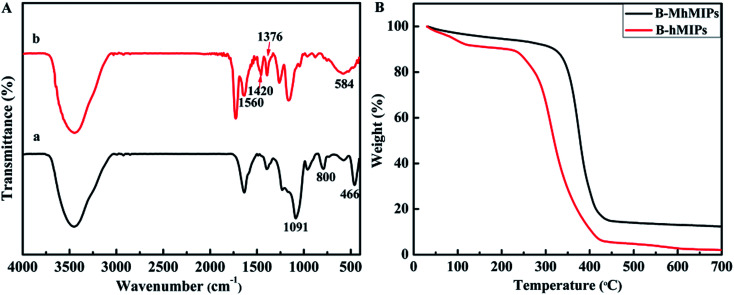
FT-IR spectra of MCM-48 and B-MhMIPs (A); TGA curves of the B-hMIPs and B-MhMIPs (B).

The FT-IR spectra of MCM-48 and B-MhMIPs are shown in Fig. 4A. In the FT-IR spectra of MCM-48 (a), the typical bands around 1091 cm^−1^ and 800 cm^−1^ resulted from the Si–O asymmetric and symmetric stretching vibrations, and the band at about 466 cm^−1^ was ascribed to Si–O bending vibration.^9^ The disappearance of the 800 cm^−1^ and 466 cm^−1^ and formation of weak bands at 1091 cm^−1^ revealed that MCM-48 was removed. Simultaneously, a series of new peaks at 1420 cm^−1^ and 1376 cm^−1^ resulted from B–O adsorption, and the peak at 1560 cm^−1^ indicated the aromatic rings stretching vibration.^31^ Moreover, the adsorption band at 584 cm^−1^ showed the successful immobilization of Fe_3_O_4_ nanoparticles.^25^

The TGA determinations (Fig. 4B) under N_2_ atmosphere were carried out to investigate the thermal stability of B-hMIPs and B-MhMIPs, and the experiment was performed from 20–700 °C at a ramp rate of 10 °C min^−1^ under N_2_. Thermal gravimetric analysis revealed an obvious weight loss for B-hMIPs in the temperature range 230–420 °C, and 98% of its quality was lost at 700 °C. Likewise, the maximum weight loss occurred in the range 320–430 °C of B-MhMIPs, and 85% of its quality was lost at 700 °C. These results indicated that B-MhMIPs possess higher thermal stability for analytical applications below 300 °C.

### Optimization of the adsorbents procedure

The performance of the prepared B-MhMIPs adsorbents was evaluated. Various experimental parameters including sample pH, adsorbent amount, sample volume and adsorption time were studied and the optimized conditions were pH as 4.0, amount of adsorbent as 20 mg, sample volume as 10 mL, adsorption time as 40 min (Fig. S2†).

### Selectivity of the adsorbents

**Fig. 5:**
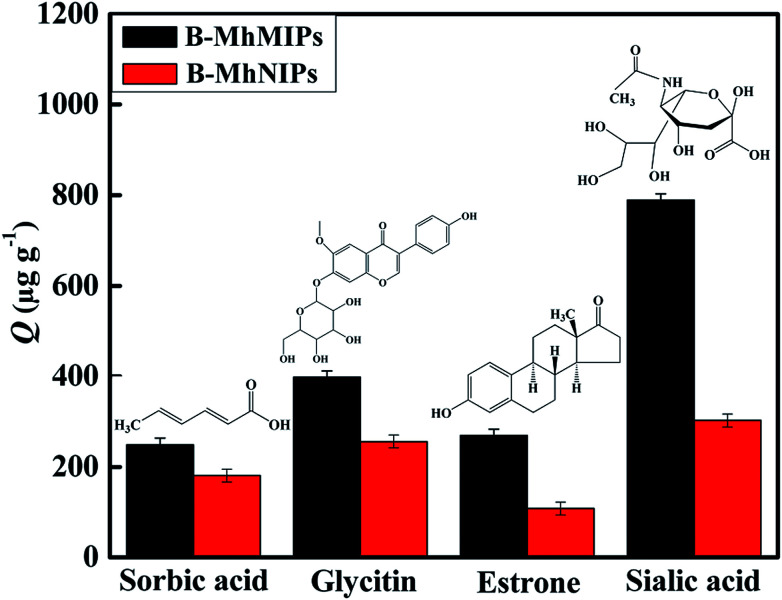
The selectivity adsorption capabilities of B-MhMIPs and B-MhNIPs for different compounds (*n* = 3) and structures of sorbic acid, glycitin, estrone and sialic acid.

To evaluate the selectivity of adsorbents, three reference compounds: sorbic acid, glycitin and estrone (the structures of these compounds are shown in Fig. 5) were selected to detect the selectivity adsorption of B-MhMIPs/B-MhNIPs under the same adsorption conditions. The main difference between SA and sorbic acid/glycitin/estrone is as follows: sorbic acid and estrone with non-containing *cis*-diol groups and the structures are different from SA; glycitin containing the *cis*-diol groups but the structure is different from SA as well. The results (Fig. 5) indicated that the adsorption amount of B-MhMIPs for SA was 788.93 μg g^−1^, which was 2.61 times that in B-MhNIPs, suggesting satisfactory selectivity recognition of the B-MhMIPs for SA. B-MhMIPs has poor adsorption capability for sorbic acid, its adsorption amount was 249.08 μg g^−1^, which was 1.38 times that in B-MhNIPs, and the reason are the greatest difference of structure and molecular-interaction between SA and sorbic acid. Additionally, the existence of straight chain structure of sorbic acid, which presumably prevented it from entering into the imprinting cavities due to steric effect. On the other hand, B-MhMIPs had moderate selectivity for glycitin, its adsorption amount was 397.84 μg g^−1^, which was 1.56 times that in B-MhNIPs. The size difference between glycitin and SA was the main cause for the adsorption capacity gaps between the two compounds, even though they are all contained *cis*-diol structure. The reference compound of estrone, non-containing *cis*-diol groups, with different structure and chemical properties, was less adsorbed by both of B-MhMIPs (269.20 μg g^−1^) and B-MhNIPs (107.68 μg g^−1^). Comparatively, the B-MhNIPs nanoparticles has selective recognition sites to *cis*-diol-containing groups by APBA as a functional monomer, showing the certain (almost the same) extraction performance toward *cis*-diol-containing compounds (SA and glycitin). However, the B-MhMIPs exhibited remarkably higher adsorption capacity for the SA molecule than glycitin, which further proved that imprinted cavities do not exist on the surface of B-MhNIPs. In consideration of overwhelming superiority of B-MhMIPs, it was chosen as the adsorbent material in the following studies.

### Reproducibility

The reproducibility of the preparation and the practicability of the extraction materials are crucial factors, which ensure the robustness of the extraction method. The intra-batch relative standard deviation (RSD) of the SA calculated by five extractions of targets using five B-MhMIPs synthesized in one batch, was found to be 1.6%. The inter-batch RSD, calculated by extractions of SA using five B-MhMIPs synthesized in different batches, was 5.5%. The results demonstrated good reproducibility and practicability of the B-MhMIPs.

### Evaluation of the method

Under the optimized conditions, the method was applied for the determination of SA in rabbit serum samples. The calibration curve was constructed by plotting peak area *versus* concentration. The regression coefficients (*R*^2^) value was determined to be 0.9963. The limit of detection (LOD) and limit of quantification (LOQ) were calculated based on the S/N ratio of 3 and 10, and the data were 0.025 μg mL^−1^ and 0.083 μg mL^−1^, respectively. The intra-day and inter-day precisions of the method were respectively calculated as 0.9% and 1.4% (*n* = 3), indicating that the method has high sensitivity and reproducibility.

In addition, the B-MhMIPs method was compared with those previous methods reported for the detection of SA. These important results including the solvent volume, LOD, LOQ, and linear ranges are listed in Table 1. The solvent volume of the proposed method was almost the same with that reported by ref. 29, and less than that of ref. 32. Although the sample volume was larger than the other two methods,^33,34^ this method was performed using the principles of magnetic solid phase extraction, adsorbents were separated by magnet in a short time. Clearly, the B-MhMIPs contained a hollow porous structure and a high capacity with amounts of recognition sites, can ensure accessibility of SA to almost all the sites. Therefore, the B-MhMIPs as selective adsorbent materials have overwhelming superiority for the selective extraction of SA for further test. Moreover, the LOD value of the proposed method is lower than that of most of the previous methods, as a new material, the B-MhMIPs is an ideal candidate as magnetic adsorbent.

**Table 1:** Comparison of different sample preconcentrations and detection methods for the determination of SA

Application	Pretreatment	Detection method	Solvent volume^d^ (mL)	LOD (μg mL^−1^)	LOQ (μg mL^−1^)	Linear range (μg mL^−1^)	Ref.
MFGM^a^	—	HPLC	8 mL	10.0	21.0	50–500	29
Infant formulas	—	Spectrophotometric	100 mL	0.29 mg L^−1^	0.97 mg L^−1^	6–150	32
Biological samples	—	Scanning potentiostat	20 μL	10 μmol mL^−1^	—	—	33
Human serum	UCSHD^b^	HPLC-MS/MS	—	1.08 ng mL^−1^	3.59 ng mL^−1^	0.5–16 μmol mL^−1^	15
Human serum	DSPE^c^	HPLC	2 mL	0.11 ng mL^−1^	0.36 ng mL^−1^	10–1000 pmol L^−1^	34
Rabbit serum	B-MhMIPs	HPLC	10 mL	0.025	0.083	0.5–10	This work

a Yak milk fat globule membrane.

b Ultrasonic-assisted closed in-syringe hydrolysis and derivatization.

c Dispersive solid phase extraction.

d Organic solvent volume in the pretreatment step.

### Analysis in real samples

**Fig. 6:**
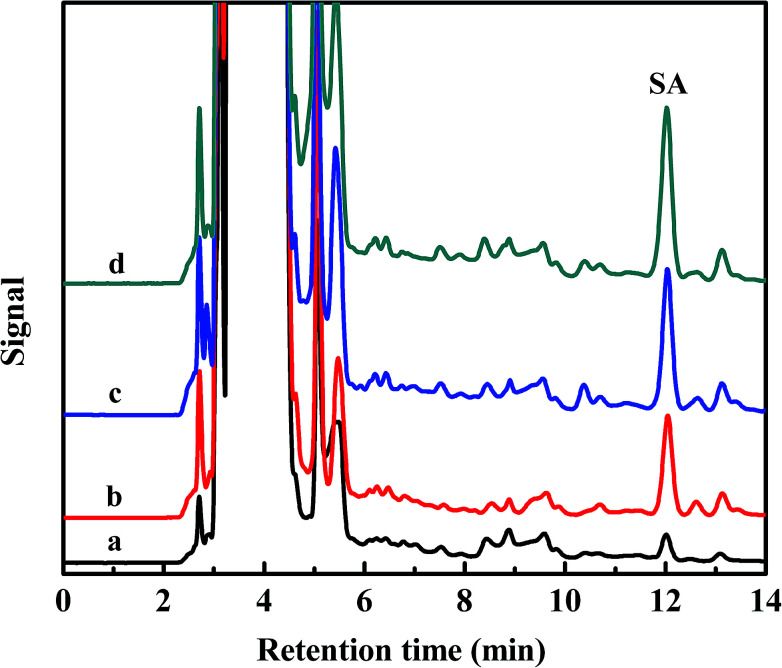
Chromatograms of serum diluted 50-fold (a), sample spiked with 0.5 μg mL^−1^ (b), sample spiked with 1.0 μg mL^−1^ (c) and sample spiked with 2.0 μg mL^−1^ (d).

The serum samples were spiked with SA standard solutions at three concentration levels (0.5 μg mL^−1^, 1.0 μg mL^−1^ and 2.0 μg mL^−1^) to assess the matrix effects, respectively. pH of all solution under test were controlled at 4.0. Fig. 6 shows the chromatograms of serum diluted 50-fold (a), sample spiked with 0.5 μg mL^−1^ (b), sample spiked with 1.0 μg mL^−1^ (c) and sample spiked with 2.0 μg mL^−1^ (d) (eluted with 20 mmol L^−1^ pH 10.0 phosphate buffer solution).

The recoveries and precisions of the B-MhMIPs SPE method were calculated. The recoveries were in the range of 70.9–106.2% with the RSD less than 2.8%, which were shown in Table 2. These results demonstrated that the prepared B-MhMIPs were selective adsorbents for the extraction of SA in rabbit serum, which can provide some guidance for the determination and separation of SA with low concentrations in real samples.

**Table 2:** Recoveries (%) of SA in real samples (*n* = 3)^a^

Sample			SA
Rabbit serum 1	Detected (μg mL^−1^)		20.0
	Recovery ± RSD (%)	Level 1	106.2 ± 0.2
		Level 2	81.2 ± 0.3
		Level 3	73.6 ± 0.3
Rabbit serum 2	Detected (μg mL^−1^)		24.4
	Recovery ± RSD (%)	Level 1	100.0 ± 1.3
		Level 2	70.9 ± 0.7
		Level 3	86.0 ± 2.8
Rabbit serum 3	Detected (μg mL^−1^)		24.7
	Recovery ± RSD (%)	Level 1	98.5 ± 0.5
		Level 2	75.0 ± 2.7
		Level 3	97.3 ± 0.1

a Level 1: 0.5 μg mL^−1^; level 2: 1 μg mL^−1^; level 3: 2 μg mL^−1^.

## Conclusion

In this work, the B-MhMIPs were successfully synthesized and exhibited a satisfactory magnetism, higher binding capacity and faster adsorption rate owing to the Fe_3_O_4_ nanoparticles, hollow structure, molecularly imprinted sites and boronate affinity, and the adsorption capacity of the B-MhMIPs is much better than B-MhNIPs. A series characterizations all showed the good performance of B-MhMIPs. In addition, the selectivity and application of B-MhMIPs proved the high sensitivity and stability in the complex biological environment in practice. Moreover, this study also proved the effect of boronate affinity can strongly depend on the experimental conditions, especially the pH value. Therefore, it is a greatly efficient and convenient discrimination approach to isolate SA from numerous *cis*-diol-containing compounds and glycoproteins through adjusting environmental pH.

## Conflicts of interest

There are no conflicts to declare.

## Supplementary Material

RA-009-C9RA00511K-s001
