# Supplementary material for: Determination of sialic acid in serum samples by dispersive solid-phase extraction based on boronate-affinity magnetic hollow molecularly imprinted polymer sorbent
Source: RSC Adv. 2019 Feb 12;9(10):5394–401. doi: 10.1039/c9ra00511k (PMC9060700; doi:10.1039/c9ra00511k)
Supplement: RA-009-C9RA00511K-s001 [file RA-009-C9RA00511K-s001.pdf]

Electronic Supplementary Material (ESI) for RSC Advances.
This journal is © The Royal Society of Chemistry 2019

Support Information

Determination of sialic acid in serum samples by dispersive solid-phase extraction based on boronate-affinity magnetic hollow molecularly imprinted polymers sorbent

Wei Huang, Xingyu Hou, Yukui Tong, Miaomiao Tian*

Key Laboratory of Photochemical Biomaterials and Energy Storage Materials, College of Chemistry and Chemical Engineering, Harbin Normal University, Harbin, 150025, China. E-mail: mmttqqq@163.com (MM Tian)

Materials and Methods

Preparation of MCM-48

MCM-48 was synthesized based on the previous report described by Schumacher et al.[1] Firstly, 2.6 g CTAB was dissolved in the 120 mL DDW, followed 4 mL TEOS and 80 mL EtOH were added into the above solution and the mixtures were stirred under room temperature for 30 min. Afterwards, 12 mL $NH_3 \cdot H_2O$ was added dropwise with continued stirring for another 6 h. Then, MCM-48/CTAB nanoparticles were collected by filtration and rinsed. The MCM-48 was obtained by calcination to remove CTAB at 823 K (2°C min^{-1}) for 6 h.

Results and discussion

Optimization of the adsorbents procedure

The performance of the prepared B-MhMIPs adsorbents was evaluated. Various experimental parameters of adsorbents including sample pH, adsorbent amount, sample volume and adsorption time were studied and optimized as follows.

Effect of adsorption pH

The adsorbents were used to adsorb SA under different pH from 2.0 to 10.0. As it is shown in Fig. S2A, the adsorption capacity achieved the maximum at pH 4.0. When the pH is too high, the adsorption capacity decreased gradually. This strategy was found by Otsuka and co-workers that SA exhibit prominent high binding capacity with common boric acid ligand when the pH is lower than the p*K*a value of the boronic acid,[2] and it can be explained as follows (Fig. S1). The structure of SA is a stable conformation due to the intramolecular hydrogen bonding. When the surrounding turned to lowered pH, the amide NH or CO located at C-5 of SA molecule can form an intermolecular B-N or B-O linkage, which was stable existence.[2] In fact, the intramolecular B-N and B-O linkages have been found to be effective strategies for the design and synthesis of boronic acids that can bind to cis-diol compounds at lower pH.[3] However, the boronic acid group will combine with hydroxyl groups on C-8 and C-9 of SA molecules under higher pH. This conformation, with higher energy,

destroys the original hydrogen bonding of SA molecules, and is not as stable as the conformation under lower pH. In addition, the chemical bonds between SA and boronic acid groups can be broken under the alkaline condition.[4] Therefore, the pH 4.0 was chosen as optimized adsorption pH.

(Embed Fig. S1)

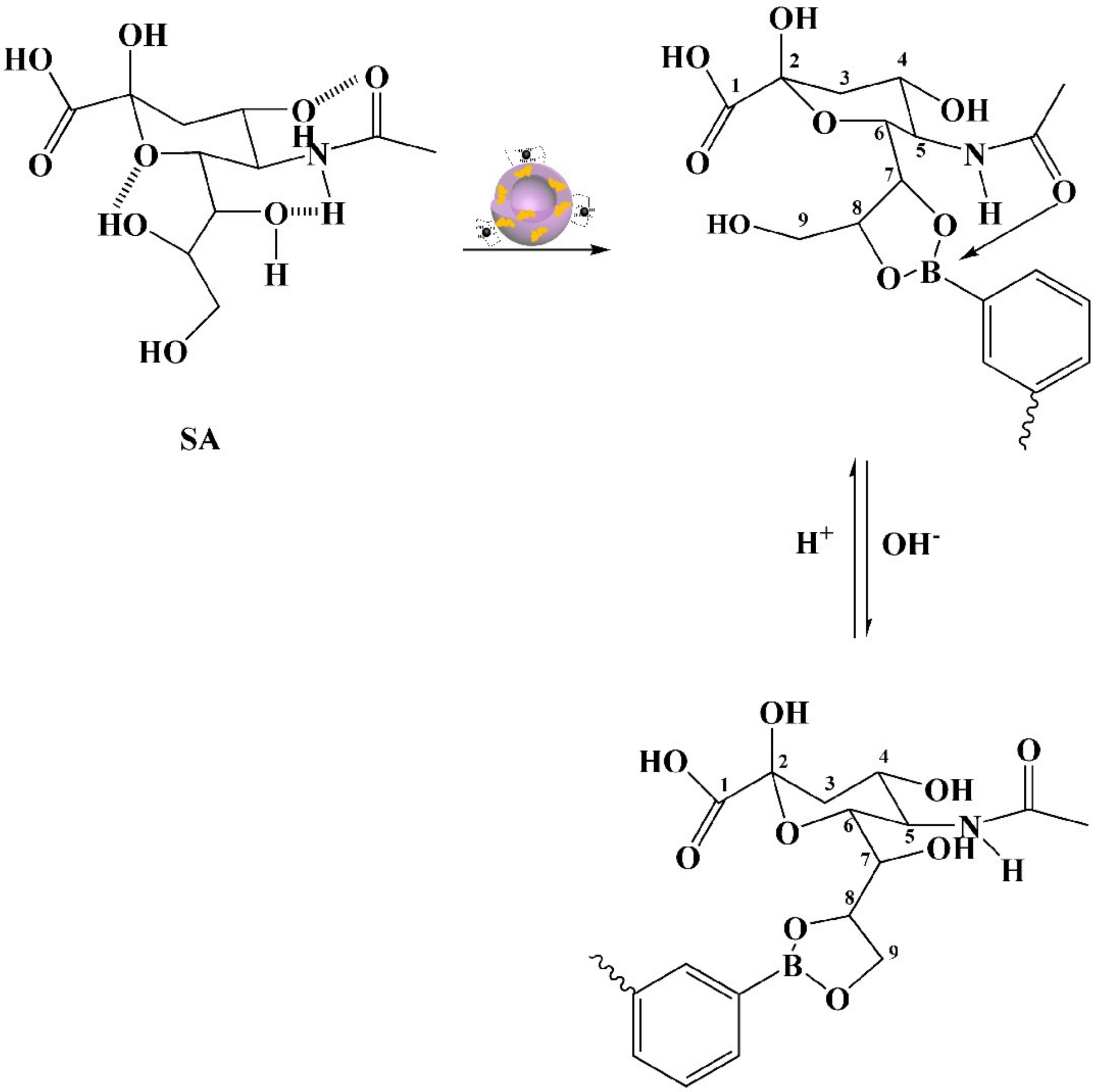

Effect of the adsorbent amount

To optimize the amount of B-MhMIPs for the exaction of SA, the range of 10–40 mg adsorbents were evaluated. It can be seen from Fig. S2B, 20 mg B-MhMIPs have the highest adsorption capacity. However, the capacity kept nearly constant in the range of 20–40 mg, indicating the remarkable exaction ability of the adsorbents. Thus, 20 mg of B-MhMIPs was used as the suitable adsorbent amount.

Effect of sample volume

The sample volume is one of key factors to affect the adsorption ability for analysis at low concentrations. The values of volume were investigated in the range of 2–10 mL. As shown in Fig. S2C, the curve rises continuously. Therefore, 10 mL sample volume was selected in the present work.

Effect of adsorption time

When 20 mg B-MhMIPs adsorbents were used for the adsorption of SA, effects of the adsorption time were constructed by varying the adsorption time in the range of 10–60 min before phase separation. As shown in Fig. S2D, the adsorption of SA with B-MhMIPs reached equilibrium within 40 min, after which the adsorption amount almost kept constant. Finally, the optimized adsorption time was chosen as 40 min.

(Embed Fig. S2)

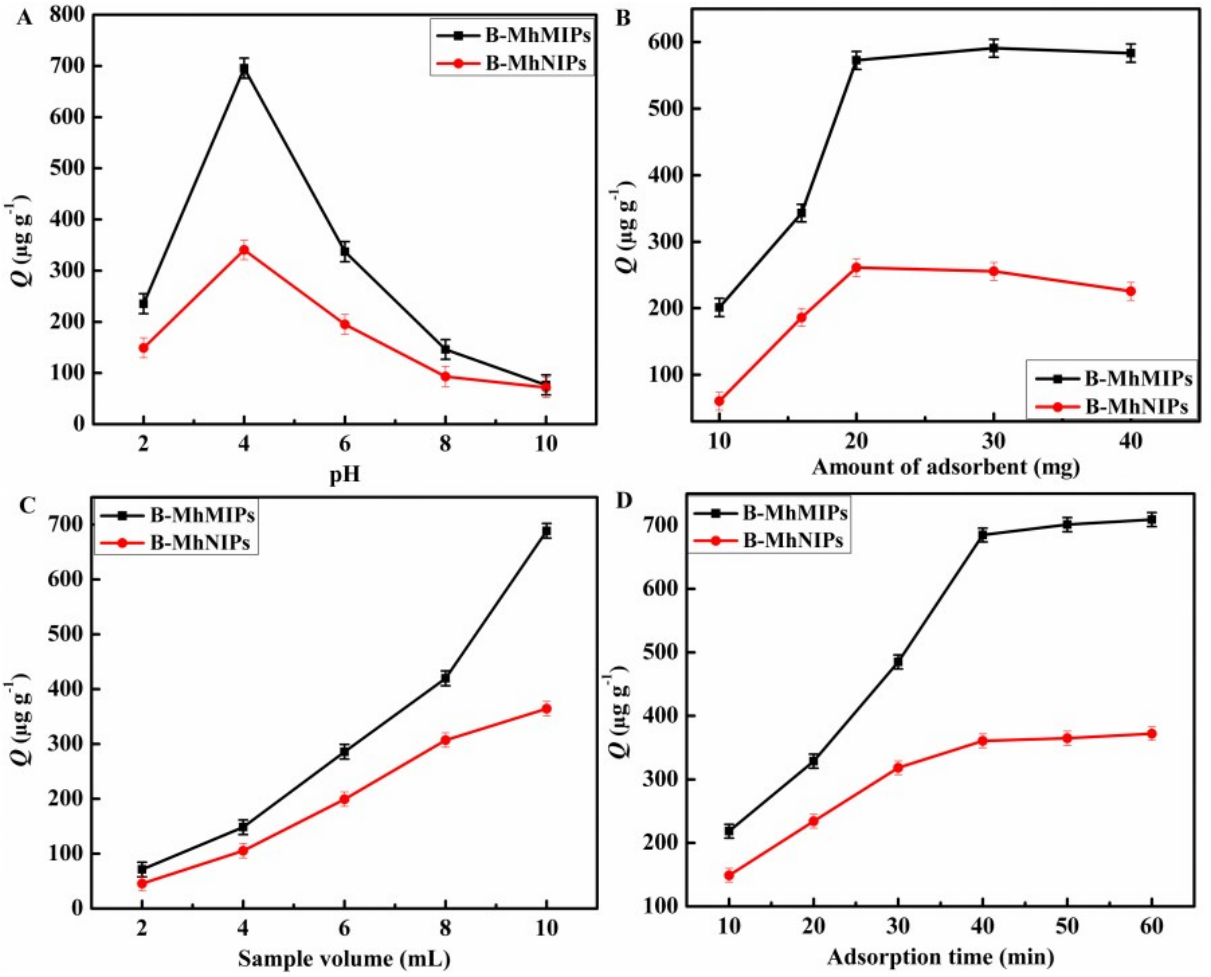

References

1 K. Schumacher, P. I. Ravikovitch, A. D. Chesne, A. V. Neimark and K. K. Unger, *Langmuir*, 2000, **16**, 4648−4654.

2 H. Otsuka, E. Uchimura, H. Koshino, T. Okano and K. Kataoka, *J. Am. Chen. Soc.*, 2003, **125**, 3493−3502.

3 Y. Lu, Z. J. Bie, Y. C. Liu and Z. Liu, *Analyst*, 2013, **138**, 290−298.

4 W. Wang, M. F. He, C. Z. Wang and Y. M. Wei, *Anal. Chim. Acta*, 2015, **886**, 66−74.
